# Reliability Evaluation Method for Array Antenna Considering Performance Changes

**DOI:** 10.3390/s24061914

**Published:** 2024-03-16

**Authors:** Xinxin Huang, Sai Zhu, Guanhui Liang

**Affiliations:** Department of Electronic and Optical Engineering, Shijiazhuang Campus, Army Engineering University of PLA, Shijiazhuang 050003, China; sjzhxx2023@aeu.edu.cn (X.H.); lianggh@aeu.edu.cn (G.L.)

**Keywords:** array antenna, reliability evaluation, performance, subarray

## Abstract

The existing array antenna reliability evaluation method based on the n/k system is analyzed. As the failed T/R module’s influence on the array antenna’s performance is not considered, the reliability of the array antenna is overestimated. To improve the accuracy of the array antenna reliability evaluation, the performance changes caused by T/R failures in different locations are considered. The reliability evaluation method considering the performance changes is established. The performance and probability of the array antenna’s state are calculated, and accurate reliability is obtained by calculating all the available state’s probabilities. The complexity of the reliability evaluation method is analyzed, and the reliability evaluation method for large-scale array antennae is established. The large-scale array antenna is divided into several subarrays. The performance and reliability of each subarray are analyzed, and the array antenna’s reliability is calculated through subarrays. The array antenna’s performance changes are considered with the proposed method, the overestimation problem of the existing reliability evaluation method is solved, and the accuracy of the array antenna reliability evaluation is improved.

## 1. Introduction

An array antenna has characteristics such as high power, high gain, and fast beam scanning, and it is widely used in communication systems [1,2,3], radar equipment [4,5] and other applications [6,7,8]. A phased array antenna is composed of a large number of transceiver channels, which consist of a transmitter and receiver module (T/R module) and element. The T/R module mainly completes the amplitude and phase adjustment of the transmitted and received signals. The fault of transmitting and receiving channels and the coupling [9] between different channels are important problems in the application of array antennas. When a fault occurs in the transceiver channel, such as the T/R module being faulty, the array antenna’s performance will be changed. The array antenna’s performance could decline with a large number of transceiver channel faults [10,11], and the expected function of the array antenna could be affected seriously. Lots of array antennas with high reliability have been designed [12,13,14,15].

Reliability is an important factor for the array antenna [16,17,18], and many reliability models have been proposed. The basic reliability model used for array antenna is the “K-out-of-N” model [19,20]. If the single T/R module in the array antenna is a failure, only the signal radiation of the connected element is affected. The performance of the other transceiver channel is unchanged. When a small number of transceiver channels fail, the array antenna’s performance will be slightly reduced. Therefore, the array antenna is a typical “K-out-of-N” system; that is, the array antenna’s total transceiver channels are noted as *n.* When *k* transceivers are working properly, the array antenna’s expected function can be completed. The transceiver’s reliability function is noted as *r*(*t*), and the array antenna’s reliability function is [19]

(1) $$R(t)=\sum_{i=k}^{n}C_{n}^{i}r{\left(t\right)}^{i}{(1-r(t))}^{n-i}.$$

The array antenna’s mean time to failure (MTTF) is noted as *θ*, and the array antenna’s average lifetime is [21]

(2) $$\theta =\int_{0}^{\infty }R(t)dt.$$

In practical applications, when the number of failed transceiver channels is less than 10% of the system’s total transceiver channel number, the array antenna’s performance is considered to have no significant change, and the array antenna can be used normally. That is, *k* = 90% × *n*, and the maximum number of failed transceiver channels is *f*_max_ = 10% × *n*.

In this reliability model, only the number of failed transceiver channels is analyzed, and the location of the failed transceiver channel is not considered. When the location of the failed transceiver channel is changed, its influence on the array antenna’s performance is different. Therefore, even with the faulty transceiver channel number *f* < *f*_max_, if the faulty transceiver channels are concentrated in a certain area, the array antenna’s performance could degenerate seriously, and the requirement would not be met. Therefore, there is a reliability overestimation problem during the array antenna’s reliability evaluation using this reliability model.

The reliability model of an AESA subsystem is formed using the “K-out-of-N” reliability configuration [22]. The reliability model [23] based on the performance margin is developed for satellite-based phased array antennas, considering the performance degradation and multiple sources of uncertainties. The distribution of the uplink sum-rate is asymptotically analyzed for an LIS system, and the reliability of large intelligent surfaces has been analyzed in [24]. The reliability of the array antenna with k/n redundant structure is analyzed in [25]. The reliability of irregular subarrayed phased array antenna is analyzed in [26]. A new method that efficiently and effectively analyses the statistical performance of phased-array antenna systems using a spherical harmonics expansion approach is presented in [27].

In this paper, a reliability evaluation method of array antenna considering performance changes is proposed. The performance degeneration process caused by the transceiver channel faults is considered, and a more accurate reliability is calculated. The rest of this paper is organized as follows. In Section 2, the Reliability evaluation method considering performance changes is presented, and the reliability calculation flow considering performance changes is presented and analyzed in Section 3. The reliability calculation flow considering performance changes for larger-scale array antenna is proposed in Section 4. The proposed Reliability evaluation method of array antenna considering performance changes is simulated and verified in Section 5. Finally, conclusions are reached in Section 6. Furthermore, more suggestions are put forward for future research.

## 2. Reliability Analysis Flow Considering Performance Changes

**Figure 1:**
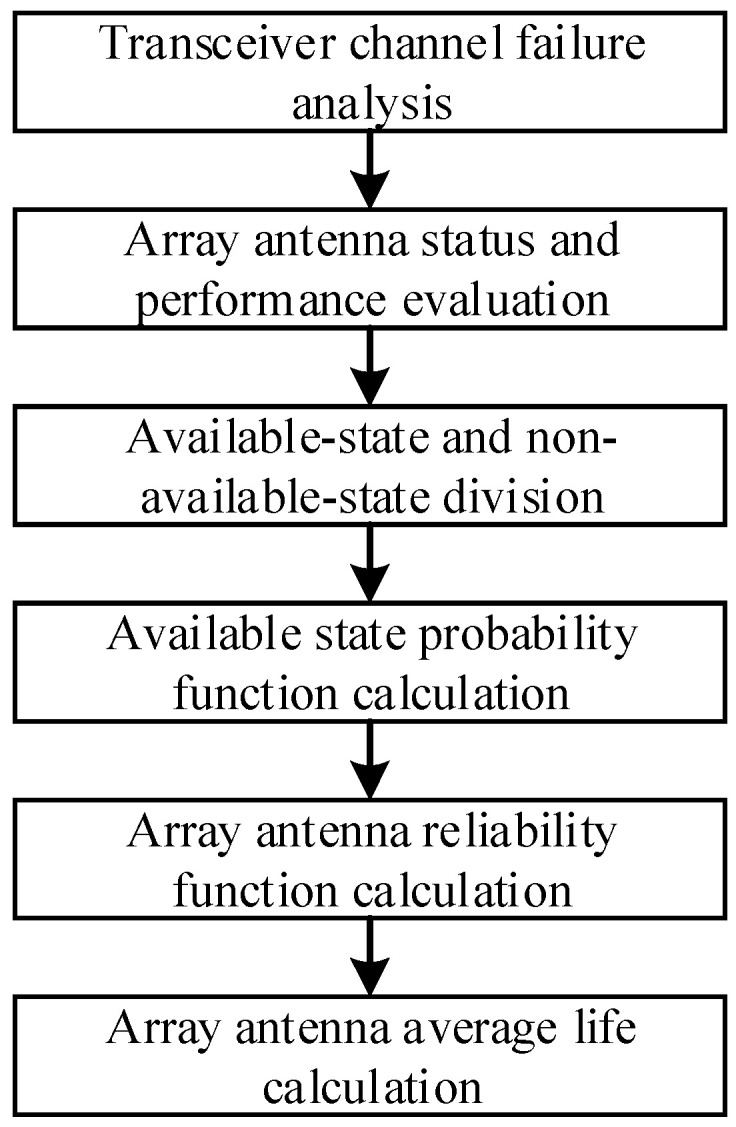
Array antenna reliability evaluation process.

To improve the accuracy of the array antenna’s reliability analysis, the performance of the array antenna in each state is calculated first. The available state and unavailable state are determined according to the performance threshold. Then the probability function of the available state is analyzed, and the reliability of the array antenna and its average life can be calculated. The steps of the reliability analysis flow are shown in Figure 1.

Six steps are included in the array antenna reliability evaluation process, as shown in Figure 1. The transceiver channel’s failure will be analyzed first. Then the array antenna’s status and performance would be evaluated. Based on the array antenna’s performance analysis, the array antenna’s state could be divided into available-state and non-available-state. Then the probability of all available states could be calculated, and the array antenna’s reliability function could be established. At last, the array antenna’s average life could be calculated.

(1) Transceiver channel failure analysis. According to the structure and composition of the array antenna, the failure characteristic of the transceiver channel is analyzed. The basis for the array antenna’s state-changing analysis is provided.
(2) Array antenna’s status and performance evaluation. According to the array antenna’s scale and structure, all possible states of the array antenna are defined. The array antenna’s performance in different states is calculated.
(3) Available-state and non-available-state divisions. According to the array antenna’s performance in each state and the performance requirements during the application process, all possible states of the array antenna are divided into available states and non-available states. In the available state, the array antenna’s performance could meet the application requirements and the array antenna can be used normally. In non-available states, the application requirement cannot be satisfied by the array antenna’s performance, and the array antenna cannot be used normally.
(4) Available state probability function calculation. According to the fault law of the array antenna’s transceiver channel, considering the transition between different states, the probability function of each available state is calculated, and the probability of the array antenna being in the available state at different times is determined.
(5) Array antenna reliability function calculation. Based on the array antenna available state probability function calculation, the array antenna availability probability is obtained by adding all the available state probabilities together. And the array antenna’s reliability function can be obtained.
(6) Array antenna’s average life calculation. Based on the calculation of the array antenna’s reliability function, the array antenna’s average life can be calculated with Formula (2).

The array antenna reliability evaluation process shown in Figure 1, evaluates each working state of the array antenna is evaluated. Each state’s performance is analyzed, and the available state is determined according to the state performance and required performance threshold. Then the probability function of each available state is calculated according to the state changing and the transition probability between different states. Based on the available state’s probability, the array antenna’s reliability function and average life can be calculated. The impact of the failure on performance is considered in the reliability evaluation process, and the array antenna’s reliability can be calculated accurately.

## 3. Reliability Calculation Flow Considering Performance Changes

### 3.1. Reliability Calculation Flow

To improve the analysis accuracy of array antenna reliability, it is necessary to accurately calculate the performance of the array antenna in each state, and determine whether the state is available according to the performance threshold. Then the available states and unavailable states can be determined, the probability of each available state is analyzed, and the array antenna’s reliability can be calculated. The steps of the reliability calculation flow are shown as follows.

(1) Failure analysis of transceiver channels

**Figure 2:**
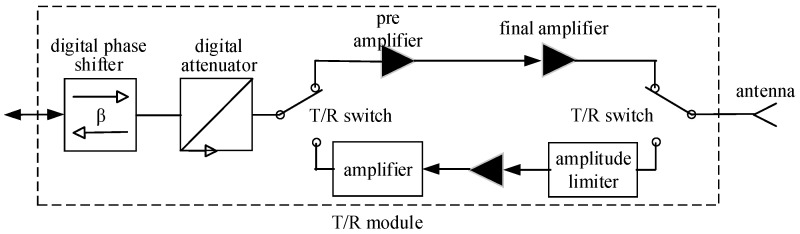
Array antenna’s transceiver channel structure.

The array antenna is composed of a large number of transceiver channels, and the structure and function of all transceiver channels are the same. The transceiver channel is composed of a transceiver module (T/R module) and radiation antenna, as shown in Figure 2.

In the transceiver channel structure shown in Figure 2, the probability of the antenna’s failure is small. The T/R module is an electronic module composed of a variety of RF devices. The probability of the T/R module’s failure conforms to the exponential distribution law, that is, its fault probability function is [21]

(3) $$F(t)=1-e^{-\lambda t},$$

where *λ* is the failure rate.

For the transceiver channel composed of the T/R module and radiant antenna, since the probability of the antenna’s failure is very small, the failure probability of the transceiver channel is equal to the T/R module’s failure probability. So the failure probability function of the transceiver channel is the same as Formula (3).

(2) Array status and performance evaluation

In the working process of the array antenna, the array antenna would be in different states, with the continuous failure of the different transceiver channels. When the array antenna is working in a different state, its performance changes too. Array antenna performance is usually represented by the antenna pattern and the parameters such as the maximum secondary lobe level, the half-power beam width, and the antenna direction coefficient.

**Figure 3:**
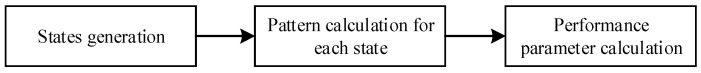
Array antenna status and performance evaluation process.

For the array antenna to be analyzed, the total number of transceiver channels is recorded as *N*, and the signal normalization amplitude of each transceiver channel is ***A*** = [*A_i_*], *i* = 0, 1, …, *N* − 1. All the array antenna’s possible states with different failure transceiver channels are traversed, and the array antenna’s parameters such as maximum secondary lobe level, half-power beam width, and antenna direction coefficient are calculated in each state. The performance of the array antenna in a certain state is evaluated with its key parameters. The calculation process is shown in Figure 3.

There are three steps in the evaluation process shown in Figure 3, state generation, direction map calculation, and performance parameter calculation:

(a) States generation. All possible states of the array antenna are generated. For the array antenna with *N* transceiver channels, the number of faults *n* = 0, 1, …, *N* − 1. Under each fault number, the corresponding total number of states is $C_{N}^{n}$. Then the total number of the array antenna in all possible states is $C_{N}^{0}+C_{N}^{1}+\cdots +C_{N}^{N}=2^{N}$. The set of array antenna’s states is denoted as *S* = {*S_i_*}, where *S_i_* is the *i*th state of the array antenna, which contains 0, 1 values with the number of *N*, when *S_i_*(*j*) = 1, it means that the array antenna’s *j*th transceiver channel is normal; When *S_i_*(*j*) = 0, it means that the array antenna’s *j*th transceiver channel is faulty.
(b) Pattern calculation for each state. For each state *S_i_*, the channel signal amplitude of ***A***.*S_i_ is regenerated according to its state. The antenna pattern in that state is calculated according to the pattern formula.
(c) Performance parameter calculation. Based on the calculation results of the antenna pattern, the array antenna’s performance parameters are calculated according to the definition of each performance parameter.

(3) Available state and non-available state division

Based on the performance evaluation of the array antenna in each state, every state is marked as an available state or non-available state, according to the application requirements of performance.

The application requirements of performance parameters are denoted as maximum secondary lobe level maxSLL_L_, average secondary lobe level avSLL_L_, half-power beam width HPBW_L_, first null beam width FNBW_L_ and direction coefficient D_L_. For the array antenna under the status *S_i_*, its maximum secondary lobe level, average secondary lobe level, half-power beam width, first null beam width and direction coefficient are denoted as maxSLL*S_i_*, avSLL*S_i_*, HPBW*S_i_*, FNBW*S_i_* and D*S_i_*. When its performance parameters meet maxSLL*S_i_* > maxSLL_L_, avSLL*S_i_* > avSLL_L_, HPBW*S_i_* < HPBW_L_, FNBW*S_i_* < FNBW_L_, D*S_i_* > D_L_, the state *S_i_* is an available state. If any of the parameters cannot meet the application requirements, the state is a non-available state.

The performance parameters and their thresholds are set according to the application scenarios of the array antenna.

(4) Available state probability calculation

For the available state of the array antenna, the occurrence probability of the state is calculated based on the number of faulty transceiver channels.

For the available status *S_i_*, the number of normal transceiver channels in the array antenna is *N_n_*

(4) $$N_{n}=\sum_{j=1}^{N}S_{i}(j),$$

In this state, the number of faulty transceiver channels in the array antenna is *N_f_* = *N* − *N_n_*.

At time *t*, the probability of a transceiver channel failure is shown in Equation (3), and then the occurring probability of the available state *S_i_* is

(5) $$p_{s_{i}}\left(t\right)={\left(1-e^{-\lambda t}\right)}^{N_{f}}{\left(e^{-\lambda t}\right)}^{N_{n}}.$$

(5) Array antenna’s reliability function calculation

Based on the analysis of all available state’s probability, the array antenna’s reliability function is calculated. The array antenna’s reliability function is the sum of all the available state’s probability. Note that the array antenna reliability function as *R*(*t*), then

(6) $$R(t)=\sum p_{s_{i}}\left(t\right).$$

In Equation (6), *S_i_* is all available states.

By substituting Formula (6) into Formula (2), the average life of the array antenna can be obtained.

### 3.2. Suitable Size Analysis of Array Antenna

For array antennas with size *N*, the total number of array antenna states that need to be analyzed is 2*^N^*. It can be seen that the calculation amount in the reliability evaluation process is increased exponentially with the array antenna’s size. For array antennas with different sizes, the number of states that need to be calculated is shown in Table 1.

As can be seen from Table 1, when the array antenna’s size is greater than 50, the total number of states calculated is bigger than 1.13 × 10^15^. The reliability evaluation process would be seriously time-consuming, so the method in this paper only targets small-scale array antennas with a scale of less than 50.

Because every possible state needs to be analyzed during the calculation process shown in Figure 1, the calculation amount of the array state and its performance evaluation is rapidly increased with the array antenna’s size. For small-scale array antennas, the reliability and average life can be accurately obtained by this method. For large-scale array antennae, the total number of available states is increased dramatically, the computing amount increases significantly, and the cost of computing time will be unbearable.

## 4. Reliability Calculation Flow Considering Performance Changes for Larger Scale Array Antenna

**Figure 4:**
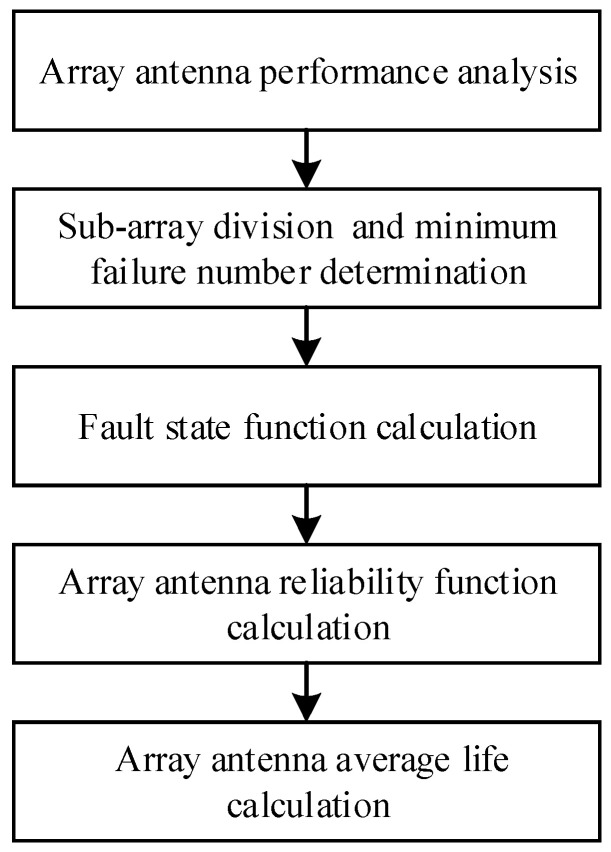
The reliability calculates flow for large-scale array antenna.

To improve the accuracy of reliability calculation, reduce the reliability calculation amount of large-scale array antenna, and improve the calculation speed, the reliability calculation flow considering performance changes for larger-scale array antenna is proposed. Based on the “K-out-of-N” reliability model shown in Equation (1), the more accurate reliability function is obtained by eliminating the fault state probability. The specific steps are shown in Figure 4.

There are five steps in the reliability calculation flow for large-scale array antennas shown in Figure 2. That is array antenna performance analysis, subarray division and determination of the minimum number of faults, fault state function calculation, array antenna reliability function and average life calculation.

(1) Array antenna’s performance analysis. The array antenna’s performance is analyzed with different faults occurring in different scale subarrays. The subarray is a small array composed of some transceiver channels in the array antenna. The basis for subarray division and determination of T/R modules’ minimum failure number is provided with the analysis result.

During the array antenna’s performance analysis, the analysis range is determined as a circle. And the center of the array antenna is taken as the center of the circle, and the fixed length *r* is taken as the radius. Within the analysis range, the fault number is set as *f* ∈ [1, *f*_max_], and the fault position is assigned randomly. Then the array antenna’s performance is calculated. In the process of performance analysis, the changes of gain and maximum sidelobe level are analyzed emphatically. In the performance calculation process of each analysis range, for the fixed failures number *f*, the performance calculation under multiple random failures can be performed, and the mean of the calculation results is taken as the performance of the analysis range *r* under the n failure number *f*.

(2) The subarray division and the determination of the minimum failure number. According to the performance of the array antenna under different analysis ranges and different fault numbers, the array antenna is divided into multiple subarrays concerning the working performance requirements. And the minimum failure number is determined. if the number of failures that occurred in the subarray is bigger than the minimum failure number, the array antenna’s performance would not satisfy the working requirement. If the array antenna can be divided into *m* subarrays, for the *i*th subarray, *I* ∈ [0, *m* − 1], its radius, T/R module number and minimum fault number are recorded as *r_i_*, *n_i_*, and *f_i_*, respectively, as shown in Table 2.

In the array antenna, the transceiver channel that is closer to center of the array antenna has a greater impact on the array antenna’s performance. For the two subarrays with the sequence numbers *i* and *j*, if the radius *r_i_* < *r_j_*, there will usually be *n_i_* ≤ *n_j_*, *f_i_* ≤ *f_j_*.

(3) Array antenna’s fault state function *F*(*t*) calculation. When the number of faulty transceiver channels *f* is less than *f*_max_, it means *f* < *f*_max_, the probability function that the array antenna is unavailable is denoted as *F*(*t*). *F*(*t*) is the sum of the failure probability of each subarray causing the array to be unavailable. Taking subarray *i* as an example, the minimum failure number that causes the array to be unavailable is *f_i_*, and then the probability function *F_i_*(*t*) that the array would be unavailable is
  (7) $$F_{i}(t)=r{\left(t\right)}^{n-n_{i}}\cdot \sum_{j=f_{i}}^{f_{\max}}C_{n_{i}}^{j}r{\left(t\right)}^{n_{i}-j}{(1-r\left(t)\right)}^{j}-r{\left(t\right)}^{n-n_{i-1}}\cdot \sum_{j=f_{i}}^{f_{\max}}C_{n_{i-1}}^{j}r{\left(t\right)}^{n_{i-1}-j}{(1-r\left(t)\right)}^{j}.$$

Array antenna’s fault state function is

(8) $$\begin{array}{cc} F(t) & =\sum_{i=0}^{m-1}F_{i}(t)\\ & =\sum_{i=0}^{m-1}\left[r{\left(t\right)}^{n-n_{i}}\cdot \sum_{j=f_{i}}^{f_{i+1}-1,f_{\max}}C_{n_{i}}^{j}r{\left(t\right)}^{n_{i}-j}{(1-r\left(t)\right)}^{j}\right] \end{array}.$$

(4) Array antenna’s reliability function and average life calculation. Based on the *k* out of *n* reliability model of Equation (1), subtract the array antenna’s fault state function, and the array antenna’s reliability function *R*(*t*) can be obtained
  (9) $$\begin{array}{cc} R(t) & =\sum_{i=k}^{n}C_{n}^{i}r{\left(t\right)}^{i}{(1-r(t))}^{n-i}-F(t)\\ & =\sum_{i=k}^{n}C_{n}^{i}r{\left(t\right)}^{i}{(1-r(t))}^{n-i}-\sum_{i=0}^{m-1}\left[r{\left(t\right)}^{n-n_{i}}\cdot \sum_{j=f_{i}}^{f_{i+1}-1,f_{\max}}C_{n_{i}}^{j}r{\left(t\right)}^{n_{i}-j}{(1-r\left(t)\right)}^{j}\right] \end{array},$$
  where *k* = 0.9*n* and *f*_max_ = 0.1*n*.

Bring Formula (9) into Formula (2), and the average life of the array antenna can be calculated.

## 5. Simulation and Analysis

### 5.1. Reliability Analysis for Small-Scale Array Antenna

Taking the Chebyshev linear array as an example, the reliability calculation is carried out by using the existing method and the proposed method in this paper. The linear array scale *N* is set to 20, the array spacing d = 0.5*λ*, *λ* is the wavelength of the radiated electromagnetic wave. And the maximum secondary lobe level SLL = −30 dB. After the array antenna’s pattern synthesis, the maximum secondary lobe level (maxSLL), mean secondary lobe level (avSLL), half-power beam width (HPBW), first null beam width (FNBW) and direction coefficient D of the array antenna under different faults are calculated.

The failure rate of the transceiver channel is set as *λ* = 4.5 × 10^−6^/h. The maximum number of faults tolerated is 2 when the existing method is used for array antenna reliability analysis. And the number of normal transceiver channels *k* is 18~20, and the array antenna’s reliability function is

(10) $$R(t)=\sum_{i=18}^{20}C_{20}^{i}{\left(e^{-4.5\times 10^{-6}t}\right)}^{i}{\left(1-e^{-4.5\times 10^{-6}t}\right)}^{20-i}$$

**Figure 5:**
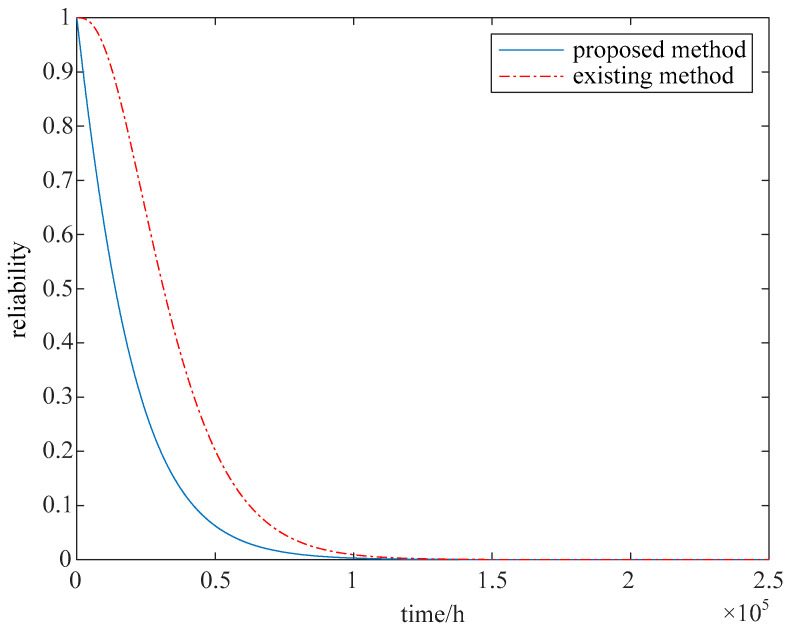
Reliability of array antenna with small-scale.

The array antenna’s reliability is analyzed with the proposed method. When the array antenna is working normally, the maximum secondary lobe level threshold is set as SLL_L_ = −21 dB. Then the 2^32^ = 1,048,576 states of the array antenna are analyzed, and the maximum secondary lobe level of each state is analyzed. All the available states are determined according to the threshold, and finally, the total number of available states is 120. The probability function of each available state is calculated using Equation (5), and the array antenna’s reliability is calculated with Equation (6). The array antenna’s reliability is evaluated with the proposed method and the existing method [19], as shown in Figure 5. When calculated using existing methods, the average life of the array antenna is 35203h. When calculated using the proposed method in this paper, the average life of the array antenna is 18,937 h.

As can be seen from Figure 5 and Table 3, the number of faulty transceiver channels was only considered in the evaluation of existing methods, and the impact of faults in different locations was not considered. For the array antenna with a scale of 20 in this paper, when the number of fault transceiver channels is 1, if the fault location is in the center of the array, the array antenna’s performance cannot meet the requirements. Although the number of failures meets the requirements, its performance does not meet the requirements, and the array antenna in this state is unavailable. In the existing reliability evaluation methods, the array antenna status is treated as non-available states only based on the number of fault transceiver channels, resulting in an overestimation of the reliability evaluation results. The available state and unavailable state are determined according to the array antenna’s performance in the proposed method, and the reliability evaluation results are more accurate.

### 5.2. Reliability Analysis for Larger-Scale Array Antenna

Taking the linear array containing 64 elements as an example, the array antenna’s reliability is analyzed with the proposed method and the existing method. The linear array is synthesized with the Chebyshev method, and the maximum secondary lobe level SLL = −45 dB. Based on the subarray’s influence on the array antenna’s performance, the array antenna is divided into four subarrays, and the minimum failure number is determined based on the influence of faulty cells on the array antenna.

When the primary–secondary lobe ratio (SLR) is considered to meet SLR < 0.5·iniSLR, that is, the primary–secondary lobe ratio drops to half of the initial primary–secondary lobe ratio, the array antenna is considered as faulty, and the minimum failure number in each subarray is shown in Table 4.

**Figure 6:**
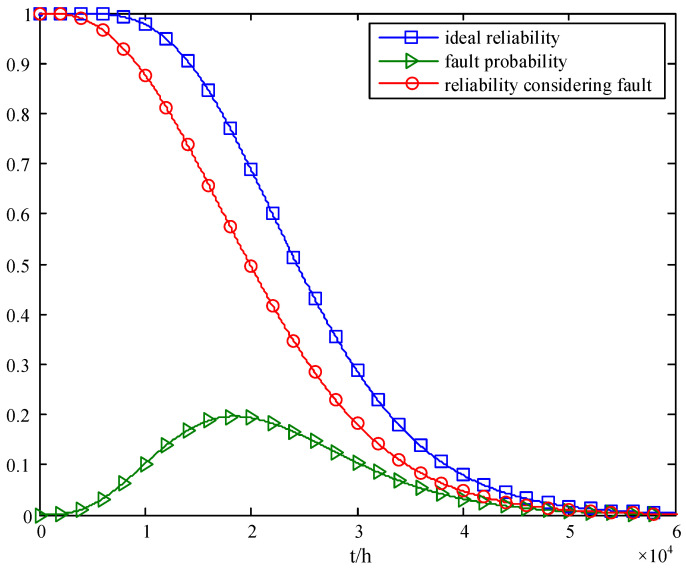
Reliability changes considering performance changes.

The failure rate of the transceiver channel is set as *λ* = 4.5 × 10^−6^/h, and the ideal reliability of the array antenna, the probability of the array antenna’s fault state, and the array antenna’s reliability considering the fault impact are analyzed by using Formulas (1), (4) and (5), respectively. And the results are shown in Figure 6 and Table 5.

As can be seen from Figure 6 and Table 5, due to the faults at different locations having different effects on the array antenna’s performance, even if the number of faults in the array antenna is less than 10%, the array antenna still has a large probability of failure. And the array antenna’s reliability calculated with the existing method is overestimated. When the array antenna’s reliability is analyzed with Equation (1), the average life of the array antenna is 25,531 h, and the average life of the array antenna is 21,158 h when considering the impact of faults on different subarrays.

Through the reliability analysis experimental results of large-scale array antenna and small-scale array antenna, it can be seen that the influence of performance changes on the array antenna’s reliability is considered in the proposed method. Each available state of the array antenna is determined accurately by analyzing the performance of all possible states, the array antenna’s reliability function is calculated based on all available states, and accurate reliability evaluation for the array antenna is achieved. For the large-scale array antenna, based on the proposed calculation process, the sub-array is further divided. The performance of each sub-array is analyzed, and the sub-array’s reliability is calculated. Then the accurate reliability evaluation for the large-scale array antenna is completed. Not only the influence of different fault locations and fault numbers on the array antenna’s performance is considered, but also the influence of the array antenna’s size on the reliability evaluation speed is considered in the proposed method. The accurate and rapid reliability evaluation for the array antenna is realized, and a novel method for the large-scale array antenna’s high-precision reliability evaluation is provided.

## 6. Conclusions

To improve the accuracy of the array antenna’s reliability analysis, a reliability evaluation process considering performance changes is established. The array antenna’s reliability is calculated through all the possible available state analyses. Through the analysis of the array antenna’s performance in every state, each non-available state is determined, the reliability overestimation problem is solved, and the accuracy of the array antenna’s reliability evaluation is improved. For the large-scale array antenna, based on the “K-out-of-N” model, the array antenna is divided into multiple subarrays, and the minimum failure number for each subarray is determined. The array antenna’s fault function is determined by each subarray. The array antenna’s reliability model considering the performance changes is obtained. The simulation results show that the influence of fault position and number on the array antenna’s performance is considered in the proposed method, the overestimation problem of existing evaluation methods is reduced and the accuracy of array antenna reliability evaluation is improved.

## Figures and Tables

**Table 1:** State number for array antenna with different sizes.

Array Antenna Scale *N*	State Number
10	1024
20	1.05 × 10^6^
30	1.07 × 10^9^
40	1.10 × 10^12^
50	1.13 × 10^15^
60	1.15 × 10^18^
70	1.18 × 10^21^
80	1.21 × 10^24^
90	1.24 × 10^27^
100	1.27 × 10^30^

**Table 2:** Subarray division.

Subarray Sequence Number	Radius *r*	TR Number in Subarray	Minimal Failure Number
0	*r*_0_	*n*_0_	*f*_0_
1	*r*_1_	*n*_1_	*f*_1_
2	*r*_2_	*n*_2_	*f*_2_
…	…	…	…
*m* − 1	*r_m_*_−1_	*n_m_*_−1_	*f_m_*_−1_

**Table 3:** Array antenna’s average life of small-scale array antenna with different methods.

Method	Array Antenna Scale	Average Life/h
existing method [19]	20	35,203
proposed method	20	18,937

**Table 4:** Subarray division for experimented array antenna.

Subarray Sequence Number	Radius *r*	TR Number in Subarray	Minimal Failure Number
0	12	24	3
1	21	42	4
2	28	56	5
3	32	64	5

**Table 5:** Array antenna’s average life of large-scale array antenna with different methods.

Method	Array Antenna Scale	Average Life/h
existing method [19]	64	25,531
proposed method	64	21,158

## Data Availability

Data are contained within the article.

## Abbreviations

T/R	Transmitter and Receiver
MTTF	Mean Time To Failure
maxSLL	maximum Secondary Lobe Level
avSLL	average Secondary Lobe Level
HPBW	Half-Power Beam Width
FNBW	First Null Beam width
